# iTRAQ-based quantitative proteomic analysis of peripheral blood serum in piglets infected with *Actinobacillus pleuropneumoniae*

**DOI:** 10.1186/s13568-020-01057-9

**Published:** 2020-07-06

**Authors:** Rining Zhu, Chuntong Bao, Baijun Liu, Jiameng Xiao, Changjiang Sun, Xin Feng, P. R. Langford, Yang Li, Liancheng Lei

**Affiliations:** 1 Key Laboratory of Zoonosis, Ministry of Education, College of Veterinary Medicine, Jilin University, Xi’an Road, Changchun, 5333 People’s Republic of China; 2 Section of Paediatrics, Imperial College London, London, UK; 3 College of Animal Sciences, Yangtze University, Jingzhou, People’s Republic of China

**Keywords:** *A. pleuropneumoniae*, iTRAQ, Porcine pleuropneumonia, Comparative proteomics, Serum

## Abstract

Porcine pleuropneumonia caused by *Actinobacillus pleuropneumoniae* (APP) is a swine respiratory disease with an important impact around the world either as a single infection or part of the porcine respiratory disease complex. The data of interaction between hosts and pathogens has becoming more crucial for exploration of the mechanism. However, up to now, comparatively little information is available on the systemic and dynamic changes that occur in pig serum in response to APP infection. This study used iTRAQ to identify differentially expressed proteins (DEPs) in pig serum in response to APP infection. Compared with the APP un-infected group (S0),there were 137 up-regulated and 68 down-regulated proteins at 24 h (S24), and 81 up-regulated and 107 down-regulated proteins at 120 h (S120). At 24 h, the immune response was not significantly enriched, but cell adhesion, cytosol, Golgi apparatus, GTP and ATP binding and regulation of cell cycle were extremely active, implying host preparation of immune response starting. Subsequently, innate immune response, negative regulation of apoptotic process, immunological synapse, adaptive immune response, the regulation of inflammatory response, positive regulation of T cell proliferation were more enhanced at 120 h then that of 24 h, representing innate immunity transferring to the adaptive, while endocytosis, cell adhesion and platelet aggregation showed obvious decline. The pathways of T cell receptor signaling pathway, cytokine–cytokine receptor interaction, complement and coagulation cascades, leukocyte transendothelial migration were active remarkably during all infection period, and more pathways could connect to form innate immune defense networks. Surprisingly, the pathways like amoebiasis, rheumatoid arthritis and malaria had been found up-regulated. As a conclusion, APP could delay host inflammatory response to the infection at early stage, and induced innate immunity to convert from adhesion, interaction into complement activation, proteasome digestion, bacterial invasion at later stage. This would increase our understanding of the porcine distinct response to APP infection.

## Introduction

*Actinobacillus pleuropneumoniae* (APP) causes porcine pleuropneumonia characterized by acute hemorrhagic fibrous porcine pleuropneumonia with respiratory distress (Sassu et al. 2018), a disease with significant impact on mortality and morbidity throughout the world (Wang et al. 2015; Yu et al. 2013). Additionally, co-infection of APP with other pathogens, such as porcine reproductive and respiratory syndrome virus (PRRSV), porcine circovirus type 2 (PCV2), swine fever virus (SFV) and swine influenza virus (SIV), are responsible for substantial economic losses in the worldwide swine industry (Wang et al. 2015; Pomorska-Mol et al. 2017; Czyzewska-Dors et al. 2017; Gottschalk and Lacouture 2014). To date, 18 serovars of App have been identified based on the composition of polysaccharides (Bosse et al. 2018). In China serovar 7 predominates followed by 4, 5, and 10 (Wang et al. 2015). Prevention and control of disease caused by APP requires understanding of the genetic and molecular mechanisms underlying interactions between the bacterium and the host (Loera-Muro and Angulo 2018). Such studies have, for example, found a complex cross talk between host epithelial cells and APP resulting in changes in bacterial gene expression including putative adhesins (Auger et al. 2009). The expression of protein in bronchoalveolar lavage fluid has been characterized by proteomics, and identified that PR-39 in BALF might have a potential as a general biomarker to determine porcine respiratory health (Hennig-Pauka et al. 2006). A proteomic reference map for pig serum proteins was finished and regarded some protein pattern changes as a prerequisite for diagnostic applications using two-dimensional electrophoresis (2-DE) (Miller et al. 2009). However, so far to our knowledge, there is no high-throughput proteomics analysis of serum after APP infection in pigs has been performed for understanding host immune defense.

A combined isobaric tag for the relative and absolute quantitation (iTRAQ)-liquid chromatography-tandem mass spectrometry (LC–MS/MS)-based proteomics approach can simultaneously quantify proteins in 4- or 8-plex samples (Zhou et al. 2017). Thus, iTRAQ-LC–MS/MS is one of the most sensitive proteomics technologies and can detect and quantitatively analyze low-abundance proteins in complex biological samples (Gajbhiye et al. 2017; Yan et al. 2017; Wang et al. 2018). An understanding of immune-related cell proliferation and differentiation in serum at different infection stages will help to reveal the immune pathogenesis of APP infection.

The immune cells and cytokines in the peripheral blood are crucial for the immune response to pathogens (Rivera et al. 2016; Reed et al. 2015). The appropriate amounts of cytokines through a variety of response pathways and inflammatory responses mediate pathogen clearance, which are self-defense mechanisms (D’Elia et al. 2013). However, the host can often be induced by APP to produce excessive cytokines, which is generally not conducive to tissue defense and repair (Bercier et al. 2019). The uncontrolled inflammatory response can cause severe illness or injury, which is even the main cause of high mortality in the host (Gomez-Laguna et al. 2014).

In this study, iTRAQ-LC–MS/MS was utilized to screen for differentially expressed proteins (DEPs) in serum collected from the peripheral blood of piglets at different infection stages. Then, the three sets of data were compared to obtain DEPs for different infection stages. We found that T cell receptor signaling pathway, cytokine–cytokine receptor interaction, complement and coagulation cascades, leukocyte transendothelial migration were active remarkably during all infection period, and more pathways could connect to form innate immune defense networks. Innate immunity converted from adhesion, interaction into complement activation, proteasome digestion, bacterial invasion at later stage. These findings might improve the understanding of the dynamics of host immune response during APP infection.

## Materials and methods

### *Actinobacillus pleuropneumoniae* preparation and experimental infection of piglets

*Actinobacillus Pleuropneumoniae* serovar 5b reference strain L20 (courtesy of Shanghai entry-exit inspection and quarantine bureau) was cultured and prepared for experimental intranasal infection studies as described previously (Bao et al. 2019). In brief, piglets were inoculated intranasally with L20 (wild type, WT) with 3.1 × 1010 CFU (Halli et al. 2014; Jiang et al. 2018). Nine, 5-week-old, APP free Landrace healthy piglets (approximately 10 kg body weight) were purchased from the animal breeding center of Jilin University. The piglets were housed in the animal room of Jilin University Veterinary Medicine School. The piglets were healthy and free from APP, as adjudged by ELISA using APP bacterial lysates as coated antigen (Halli et al. 2014). All treatments of the piglets were carried out by following animal experiments protocols approved by the State Council of People’s Republic of China.

### Sample collection and preparation

Nine piglets were randomly assigned into two groups. Group 1 (control) comprised three pigs which received PBS, and group 2 nine pigs intranasally infected with L20. Piglets were monitored for symptoms of infection as previously described (Jiang et al. 2018). Blood samples were collected in serum separation vacutainer tubes from control piglets at 0 h prior to infection, from three piglets of group 2 at 24 h post infection (mild symptoms), and from the remaining three piglets (group 2) at 120 h post infection (severe symptoms). After blood sample collection at 24 h, three APP infected piglets were randomly selected and euthanized with an overdose of sodium pentobarbital (150 mg/kg) by intravenous via ear vein, and necroscopy carried out to confirm APP infection. Blood collection from and necroscopy was also carried out on the three remaining pigs at 120 h. Serum was separated from whole blood by clotting at room temperature for 30 min, aliquots transferred into sterile cryovials and stored at − 80 °C. Depletion of albumin and IgG in thawed serum was performed using the albumin and IgG depletion kit (GE Healthcare, Watertown, MA). Immunodepleted serum was further desalted using the 2D-cleanup kit (GE Healthcare, Watertown, MA), protein dissolved in rehydration buffer (7 M urea, 2 M thiourea, 2% CHAPS, 1 mM Tris, and 1 M NaCl), and the protein concentration determined using the 2D Quant Kit (GE Healthcare, Watertown, MA). The digestion method is described as follows: After protein quantification, 100 μ g protein solution is put into centrifugal tube. Add 4 μl Reducing Reagent and react at 60 °C for 1 h. Add 2 μl Cysteine-Blocking Reagent at room temperature for 10 min. Alkylated protein solution was added to 10 K ultrafiltration tube and centrifuged for 20 min at 12,000 rpm. Add Dissolution Buffer 100 μl and centrifuge for 20 min at 12,000 rpm. Replace the new collection tube, add trypsin to the ultrafiltration tube, and react overnight at 37 °C. The next day, centrifuged for 20 min at 12,000 rpm, and the digested peptide solution was centrifuged at the bottom of the collection tube. Add 50 μl Dissolution Buffer 5 to the ultrafiltration tube, centrifuge again at 12,000 rpm for 20 min, merge with the previous step, and collect 100 μl enzymatic hydrolyzed samples at the bottom of the tube.

### iTRAQ labeling and strong cation exchange (SCX) fractionation

iTRAQ analysis was performed by Beijing Qinglian Biotech co. Ltd (Beijing, China). Post-digestion iTRAQ labeling was performed according to the manufacturer’s instructions (AB Sciex, Framingham, MA). To control for biological and technical variations, three independent iTRAQ experiments were performed. Peptides from different groups were labeled with different tags. Briefly, the peptide sample mixtures were labeled with iTRAQ reagent 114, 115, and 116. After incubation at room temperature, the peptide sample mixtures were subsequently pooled and concentrated by vacuum centrifugation. Prior to LC–MS/MS analysis, the peptide samples were purified from excess labeling reagent by strong cation exchange chromatography (SCX) using the AKTA Purifier system (GE Healthcare, Watertown, MA). Dried peptide mixture was reconstituted and acidified with solvent A (consisting of 10 mM KH2PO4 in 25% (v/v) acetonitrile) and loaded onto a Poly-SULFOETHYL column (4.6 × 100 mm, 5 μm, 200 Å, PolyLC, Inc. Columbia, MD, USA). The peptides were eluted with a 0–10% solvent B gradient (500 mMKCl and 10 mM KH2PO4 in 25% (v/v) ACN) for 2 min, 10–20% solvent B for 25 min, 20–45% solvent B for 5 min, and 50–100% solvent B for 5 min. Elution was monitored by absorbance at 214 nm, with fractions being collected every minute. Next, based on the quantity of peptides, samples were combined into 10 fractions and then desalted on C18 Cartridges (Sigma, St. Louis, MO). Each SCX salt step fraction was concentrated in a vacuum centrifuge and reconstituted with 40 μL 0.1% (v/v) trifluoroacetic acid.

### LC–MS/MS analysis

LC–MS/MS analysis was performed by Beijing Qinglian Biotech co. Ltd (Beijing, China).The peptide mixture from each fraction was injected for nano LC–MS/MS analysis. To achieve reliable statistics, three technical replicates were analyzed. Peptide mixtures were loaded onto the Thermo Easy nLC column (Thermo Finnigan, San Jose, CA) in solvent C (0.1% formic acid) and separated with a linear gradient of solvent D (80% acetonitrile, 0.1% (v/v) formic acid). The Q Exactive mass spectrometer (Thermo Finnigan, San Jose, CA) acquired data using a data-dependent top 10 method that dynamically selected the most abundant precursor ions. Determination of the target values was based on the predictive Automatic Gain Control. Dynamic exclusion was set with 60 s duration. Q-Exactive survey scans were set as 70,000 at m/z 200, and the resolution for the HCD spectra was set as 17,500 at m/z 200. The normalized collision energy was set as 30 eV, and the underfill ratio was defined as 0.1%.

### Sequence database search and protein quantification

The raw MS data from three biological replicates were transformed into Mascot generic format (.mgf) files. The MS/MS spectra were searched using Mascot (version 2.3.02; Matrix Science, London, UK) against the nonredundant International Protein Index database from the European Bioinformatics Institute. To filter the results, we employed a false discovery rate (FDR) of ≤ 1% for identification. The unique protein with at least one particular peptide was identified. The FDR was set to ≤ 1% for identification of both proteins and peptides. All peptides with a minimum confidence level of 95% (unused confidence score > 1.3) were used as qualification criteria. Relative quantification was calculated based on the ratio of peak areas from MS/MS spectra. For DEPs, a log2 fold change ≥ 1 was considered up-regulated, and a log2 fold change ≤ − 1 was classified as down-regulated. All of raw data can be found on PRIDE (https://www.ebi.ac.uk/pride/archive) with an identifier number PXD017500.

### Gene ontology annotation and KEGG

The gene ontology (GO) enrichment analysis showed enriched GO terms for DEPs among the background proteins, which connected the DEPs to the GO databases, and computed the protein numbers of each corresponding GO term. The regulation of DEPs was analyzed to find out the immune system related process. Cytoscape Clue Go was used for pathway analysis at different infection points (Bindea et al. 2009).

KEGG enrichment analysis was performed by DAVID Bioinformatics Resources 6.8 and Benjamin method was used to correct the significance of KEGG term as previous description (Huang et al. 2008, 2009; Jiang et al. 2018). (A significant difference was considered when Benjamin values < 0.05.)

### Validation of DEPs by serum ELISA

Five proteins were selected for validation by ELISA: MRC1,PRDX2,CFB,ITGB and E-Cadherin. The serum samples were allowed to clot for 1 h at room temperature and then separated by centrifugation at 2000×*g* for 15 min at 4 °C. The serum was then frozen until analyzed by ELISA. The concentration of MRC1,PRDX2,CFB,ITGB and E-Cadherin was determined using commercial porcine ELISA kits (Shanghai Jinma Biological Technology, Inc., China) according to the manufacturer’s instructions. All samples were analyzed in duplicate. Cytokine concentrations were calculated using linear regression analysis based on the OD of the cytokine standards provided by the manufacturer.

### Statistical analysis

Statistical differences were assessed by one-way analysis of variance and Dunnett’s multiple comparison tests using SPSS software (New York, U.S.). The experimental and control groups were assessed using the Student’s t-test. The minimal level of the significance was set at a P value < 0.05 with all data expressed as the mean ± SD.

## Results

### Establishment of APP experimental infection in piglets

**Fig. 1:**
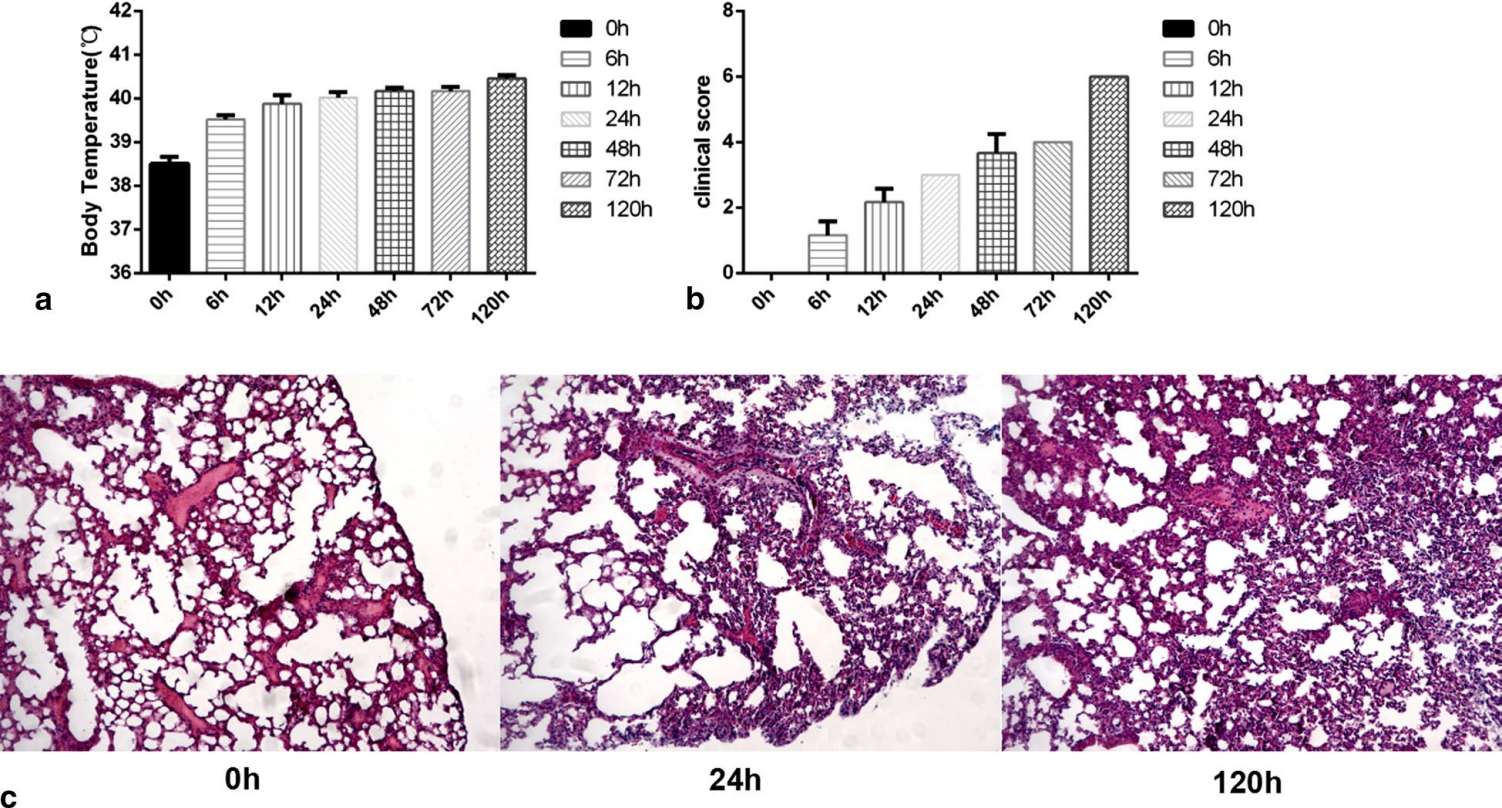
Clinical symptoms of piglets at different time points after infection

Compared with the control group (38.5 °C), all infected pigs inoculated intranasally with L20 (3.1 × 1010 CFU) had a fever, the mean rectal temperatures started to increase to 39.5 °C at 6 h post infection, and continued to go up till maximal values (40.5 °C) at 120 h after challenge (Fig. 1a). Between 48 and 120 h after challenge all infected pigs showed markedly decreased food and water consumption, rises in respiratory rate, changes in breathing patterns, unwilling movement, running noses (2 of 6 with purulent nasal discharge), and occasional coughing. At 24 h, infected piglets had mild symptoms such as small difficulty in breathing and elevated body temperature (39.7 °C). At this time point small lung lesions, areas of congestion (3/3 animals) and necrosis (1/3) were observed. The clinical symptoms score gradually increased to the maximal values at 120 h point after infection (Fig. 1b). Using X-rays to examine different periods of lung infection, shadows were found at 24 h post challenge. Thereafter, the shadow area increased, the lung texture gradually became blurred, the inflammation worsened, and it was most severe at 120 h. At autopsy three infected pigs at 120 h post infection showed a typical fibrinous haemorrhagic necrotizing pleuropneumonia, the lung lesions being readily apparent, while no lung lesions were found in the three non-infected negative control pigs.

Histological observation showed that there were a large number of inflammatory cell infiltration in the lungs of the infected group, and degeneration, necrosis and nuclear concentration of alveolar diaphragm cells took place. Lung tissue consolidation caused by the collapse of most of the alveoli, serious bleeding and congestion, exudation of plasma and cellulose in the alveoli with a large number of exfoliated alveolar epithelial cells were widely observed (Fig. 1c).

### Identification of differentially expressed genes between the different groups

All LC–MS/MS spectra were processed using Mascot software. In total, 624 proteins were identified in the serum of peripheral blood at 1% FDR using iTRAQ analysis. Using a 2.0-fold increase or decrease in protein expression as a benchmark for a physiological significant change, identified a total of 205 DEPs among the 24-h-infected group (S24) and uninfected group (S0). By comparison with the un-infected group (S0), 137 up-regulated and 68down-regulated proteins at 24 h (S24); following time passed, 81 and 107 down-regulated proteins at 120 h (S120) were identified. Comparison of the 120 h with the 24 h-infected groups, identified 58 up-regulated and 145 down-regulated proteins. Obviously, APP infection induced a severe interaction with host at early stage, but 5 days later, up-regulated genes were dramatically decreased, presenting the fighting between the pathogen and the host trended to be alleviating.

### Host cellular component, molecular function, and biological processes have changed mainly after APP infection

**Fig. 2:**
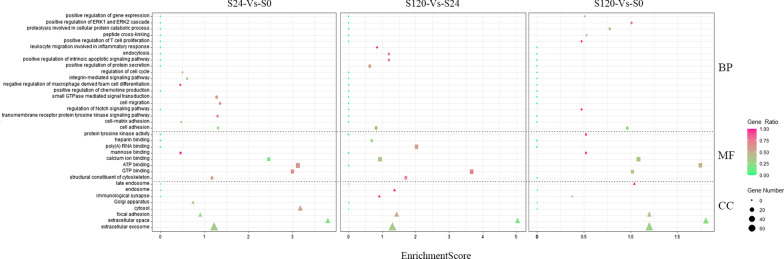
Gene ontology annotation analysis. Cellular component (CC), molecular function (MF) and biological process (BP); GO term is the X-axis and the numbers of enrichment are the Y-axis. The Q value < 0.05 were defined significant

DEPs were identified using GO for gene function classification related to the cellular component, molecular function, and biological processes. As shown in Fig. 2, gene ontology (GO) analysis of total proteins in serum can be categorized into diverse functional classes related to the cellular component (CC), molecular function (MF), and biological process (BP). Comparisons were made of up-and down-regulated proteins in the“S24-Vs-S0”, “S120-Vs-S0”, and “S120-Vs-S24” groups (Additional file 1: Tables S1, Additional file 2: Table S2, Additional file 3: Table S3, Additional file 4: Table S4, Additional file 5: Table S5, Additional file 6: Table S6) with immunologic, physiologic, and acute-phase proteins predominating. The enrichment of the DEPs classified the proteins mainly to be involved in the extracellular exosome, focal adhesion, cytosol of cellular component, ATP binding, GTP binding, structural constituent of cytoskeleton and mannose binding of molecular function and other biological processes at the “S24-Vs-S0” stage (Fig. 2 Left). The number of DEPS involved in the immune response was not significantly enriched at this stage, but cell adhesion, cytosol, golgi apparatus, GTP and ATP binding and regulation of cell cycle were extremely active, implying host preparation of immune response starting. When the infection continued to be at 120 h, immune response apparently had functioned, i.e., immunological synapse, inflammatory response, endocytosis, extracellular exosome were dramatically active by comparison to S24 (S120-Vs-S24) (Fig. 2 Middle). If compared the data at 120 h with that of uninfected group (S120-Vs-S0), it was found that the DEPs were significantly enriched in extracellular exosomes, focal adhesion, calclum ion binding,positive regulation of T cell proliferation,immunological synapse, ATP binding and positive regulation of ERK1 and ERK2 cascade (Fig. 2 Right), which also revealed innate immunity transferring to adaptive immunity. Especially, we are interested in finding that extracellular exosome went up remarkably during all infection period, and exosome maybe play an important role in anti-infection process.

**Fig. 3:**
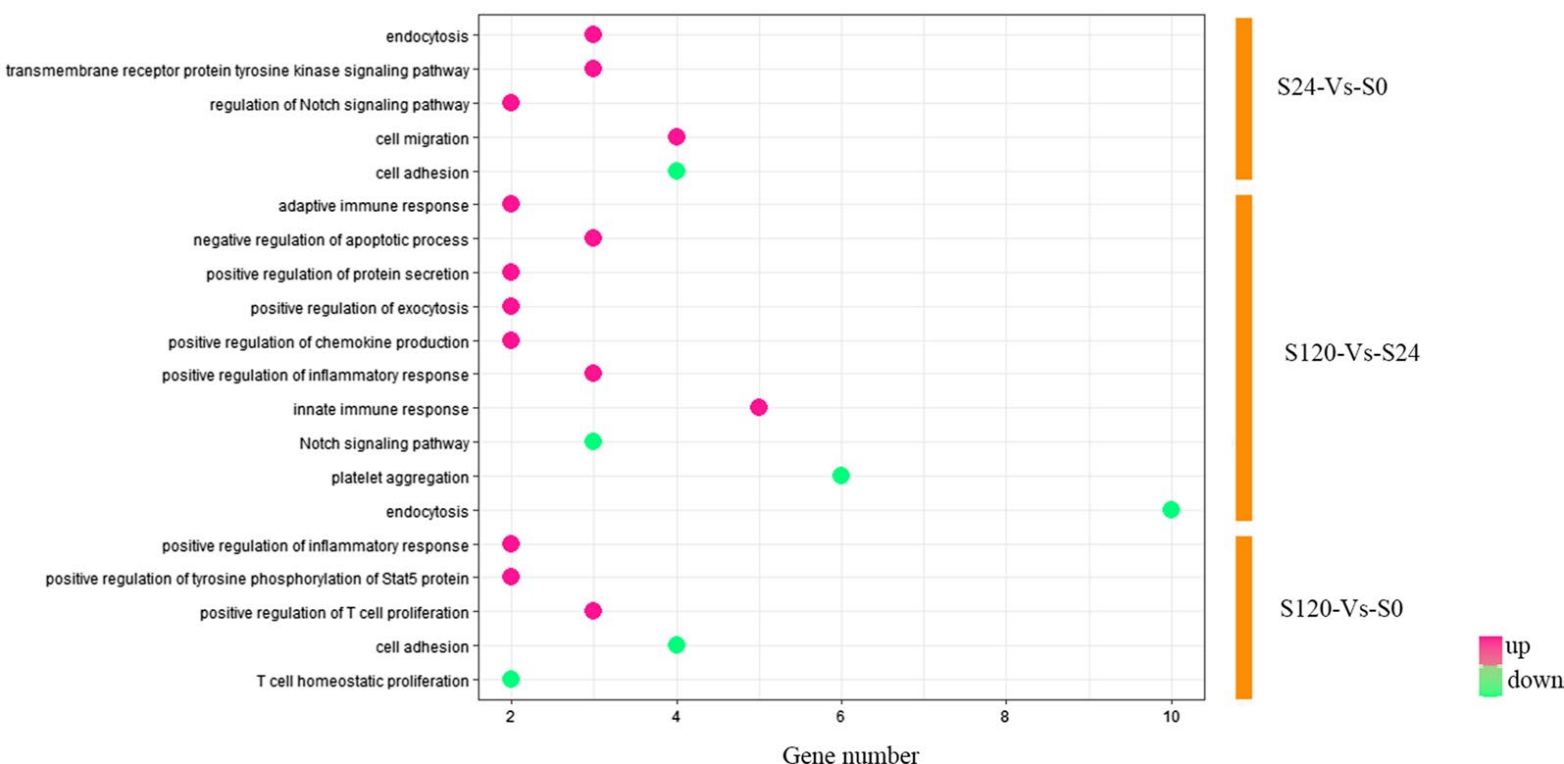
The regulation of DEPs by GO annotation analysis. GO term is the X-axis and the numbers of genes are the Y-axis. The P value < 0.05 were defined significant

Furthermore, the various processes of each stage were analyzed to identify the related immune system process in order to understand host response preferably. We also found that endocytosis, cell migration and transmembrane receptor protein tyrosine kinase signaling pathway were also involved in the response to APP infection at early stage, indicating immune clearance of APP is a focus of threat this stage. Subsequently, innate immune response, negative regulation of apoptotic process, adaptive immune response were more enhanced at 120 h then that of 24 h. However, with prolongation of infection time, up-regulated DEPs were mainly enriched in the regulation of inflammatory response, positive regulation of T cell proliferation and positive regulation of tyrosine phosphorylation of Stat3 protein, while endocytosis, cell adhesion and platelet aggregation showed obvious decline (Fig. 3).

### The pathways of cell cycle, T cell receptor signaling pathway, NF-kappa B signaling, cytokine–cytokine receptor interaction, complement and coagulation cascades were intensified during APP infection

**Fig. 4:**
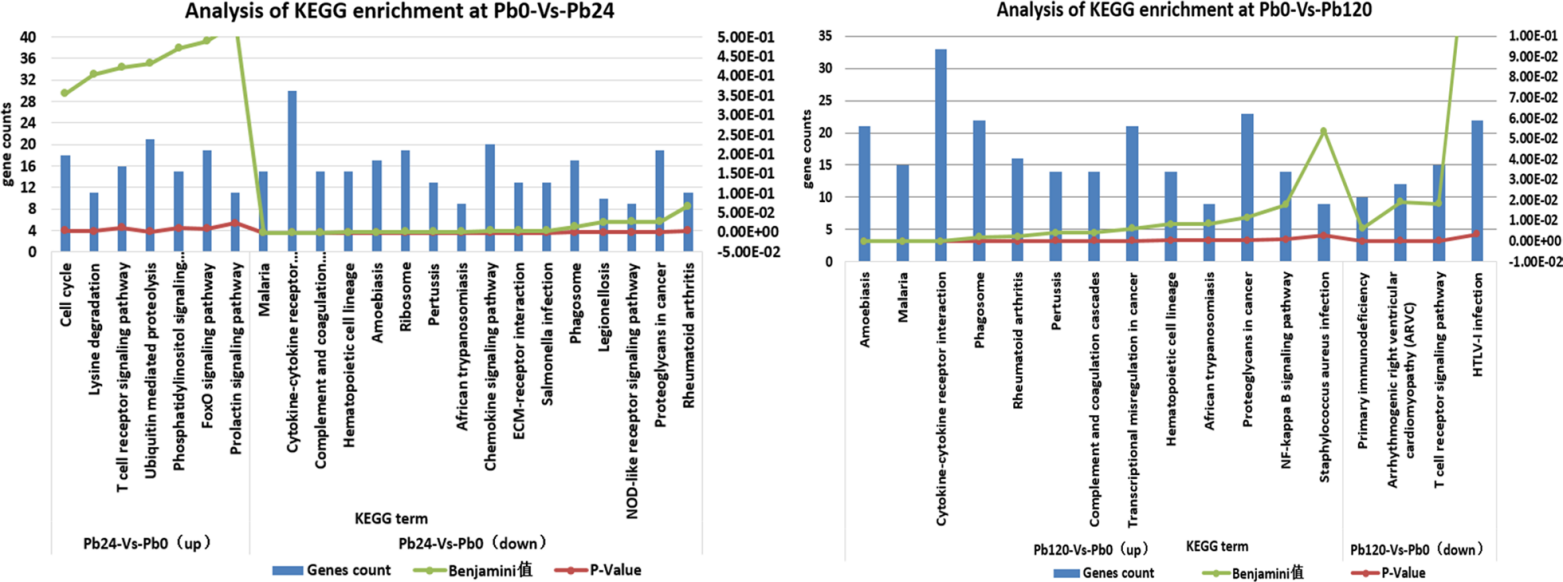
KEGG analysis. KEGG term is the X-axis and the numbers of enrichment are Y-axis. The Benjamini < 0.05 were defined significant

KEGG analysis which explored metabolic pathways and their relationship showed that the pathways related to cell cycle, lysine degradation, T cell receptor signaling pathway, ubiquitin mediated proteolysis pathway, prolactin signaling pathway and phosphatidylinositol signaling pathway at “S24-Vs-S0” stage were distinctly increased after APP infection, while cytokine–cytokine receptor pathway, chemokine signaling pathway, ECM-receptor interaction, NOD-like receptor signaling pathway, ribosome and phagosome pathway were decreased. But at “S120-Vs-S0” stage, the pathways of amoebiasis, malaria, cytokine–cytokine receptor interaction, complement and coagulation cascades, hematopoietic cell lineage, African trypanosomiasis, proteoglycans in cancer, NF-kappa B signaling pathway, staphylococcus aureus infection were extremely up-regulated, following with down-regulation of the pathways of primary immunodeficiency, arrhythmogenic right ventricular cardiomyopathy, T cell receptor signaling pathway and HTLV-I infection (Fig. 4).

**Fig. 5:**
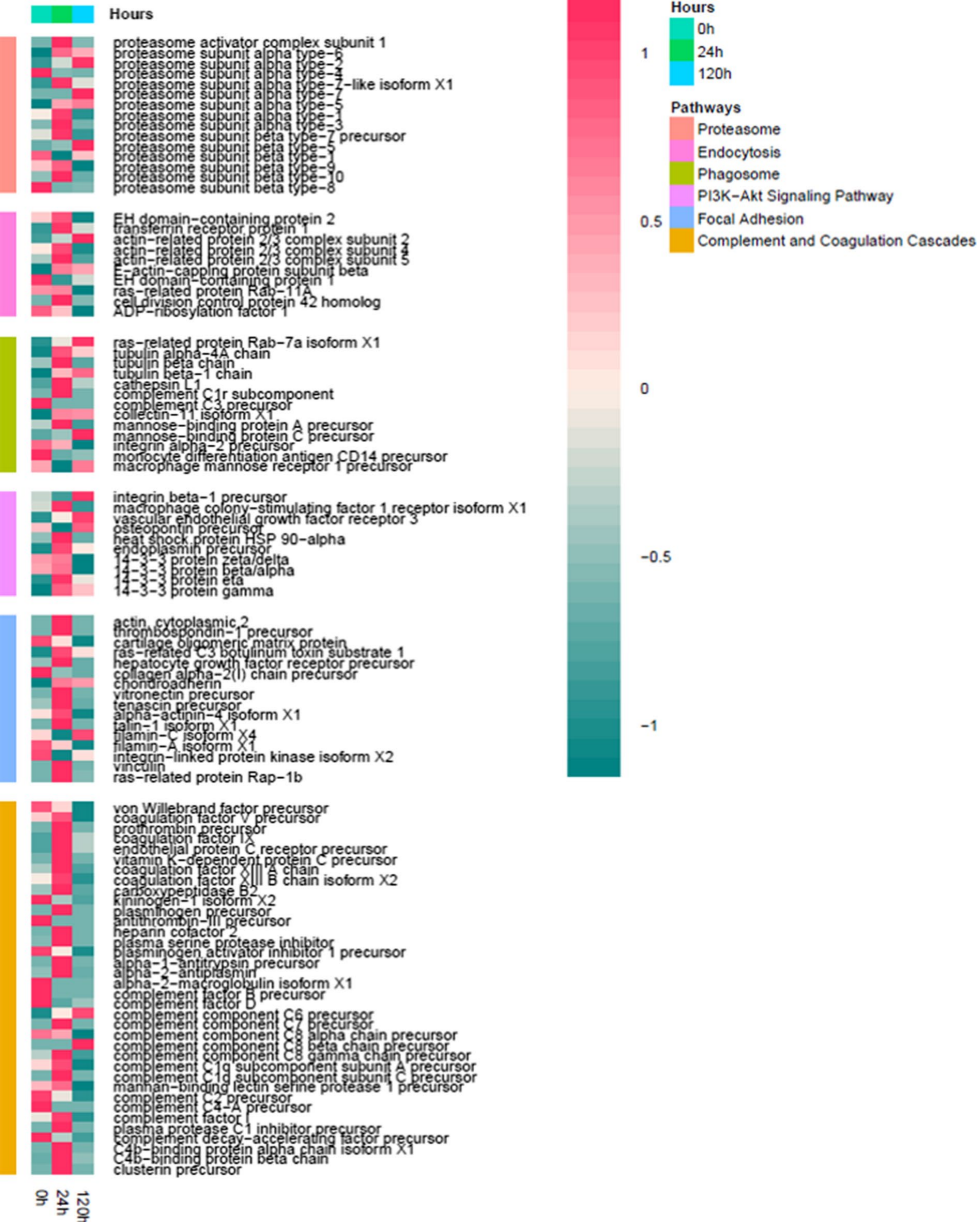
Heat map of DEPs in innate immune pathways. Time (h) is the X-axis and the DEP term is the Y-axis

Being both up and down regulations usually existing in a pathway including a number of proteins at the same time, the details of protein changes are necessary to grasp exact information associated with APP infection. So further investigation of some active pathways had been finished for better understanding of DEPs in some interesting pathway. In focal adhesion pathway which related to ECM-receptor interaction, cytokine–cytokine receptor interaction, ILK and Cdc42 were increased to promote cell proliferation and cell survival. The up-regulated RhoCdc42 and p38 in T cell receptor signaling pathway led to NFAT high expression and then positive regulation of cell proliferation and cytokine release, such as IL-2,IL-4,IL-5 release. FoxO signaling pathway promoted the apoptosis through the up-regulation of p38 and BAX. About bacterial invasion of epithelial cells, we found that Cdc42 and Arp2/3 up-regulated in Listeria infection pathway and Salmonella infection pathway, followed by ILK up-regulation in staphylococcus aureus infection pathway, jointly promoted actin production and polymerization, at last enhanced internalization and vacuole formation. In proteasome pathway, α2,β2 andβ6 proteins were highly expressed in 20S core particle to enhance its innate function. As to cell adhesion molecules, PECAM1 upregulation increased tight junction in endothelial cells and leukocyte trans-endothelial migration. MBL, C6, C7,C8 and C9 were increased significantly to promote cell lysis through complement activation and MAC formation in complement and coagulation cascades pathway. We picked up several interesting pathways to analyze their differential expression proteins (Fig. 5).

Looking at the whole infectious period, the pathways like amoebiasis, malaria, rheumatoid arthritis malaria, had been found up-regulated strangely, and it may imply there was the different interaction with host of APP from other extracellular bacteria. After the review of these pathways, we observed that APP infection activated the high expression of COL protein and EhLimA in amoebiasis pathway, leading to the upregulation of actin rearrangement and C8, and then inhibition of membrane attack complex. In malaria pathway, PECAM1 was expressed highly to enhance cell adhesion. APP infection may stimulate systemic immune disorder through upregulation of C1q, C2, C4, C8 to increase the production of MAC and the tissue injury and damage, like rheumatoid arthritis.

The changes of these pathways indicated that innate immunity was converted from adhesion, interaction into complement activation, proteasome digestion, bacterial invasion from early stage to late stage, of course, phagosome and endocytosis still continued to be as anti-infection measures. The results of the KEGG analysis were consistent with the GO analysis.

### A complicated innate immune defense network formed at later stage

**Fig. 6:**
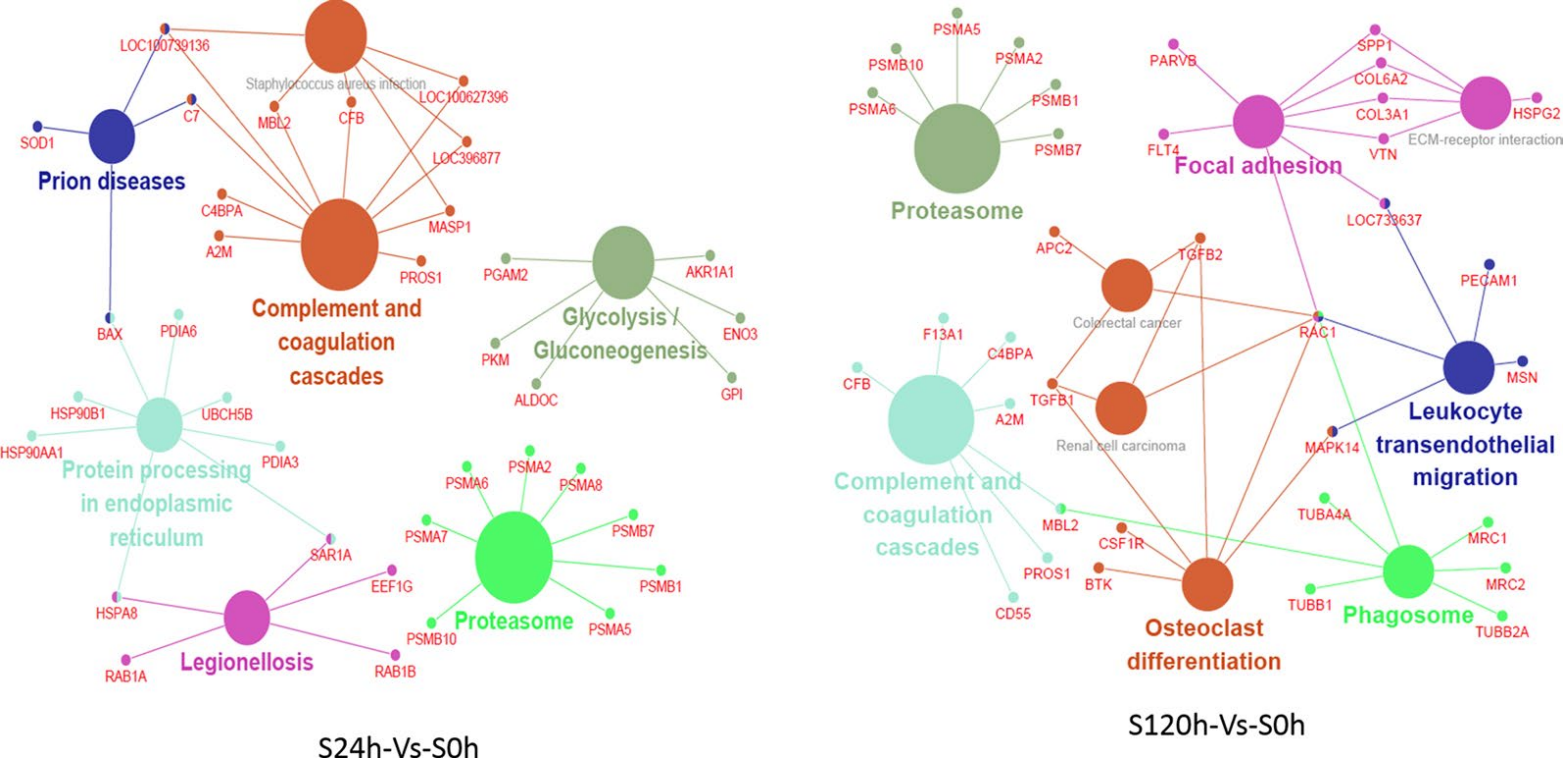
The related DEPs and pathways. Left: at “S24-Vs-S0” stage. Right: at “S120-Vs-S0” stage. The nodes represent that had cross-talk between each other

Pathway and network analysis identified complement and coagulation cascades as playing an important role in the process of the porcine host response to APP (Fig. 6). We analyzed the relationship between DEPs and signaling pathways at different infection stages. These cross-talk proteins associated with two or more pathways were noteworthy. The network was subsequently imported into Cytoscape for enrichment analysis. In the early stage of infection, MASP1, MBL2, CFB, loc396877, loc100627396 linked complement and coagulation cascades with Staphylococcus aureus infection pathway, and C7, loc100736136 linked the Staphylococcus aureus infection with the prion disease pathway. In addition, HSPA8 and SAR1A connected the protection processing in endoplasmic reticulum with legionellosis pathway. Therefore, an innate immune defense network had been formed by the pathways of complement and coagulation cascades, Staphylococcus aureus infection, protein processing in endoplasmic reticulum, prion disease and Legionellosis, being as the core knots of DEPs. Some DEPs form independent networks around the pathways of glycolysis/glycogenesis and proteasome, respectively.

In the later stage of infection, focal adhesion pathway was associated with ECM receptor interaction pathway through SPP1, col6a2, COL3A1 and VTN proteins, while focal adhesion pathway linked with leukocyte transendothelial migration pathway by RAC1 and LOC733637. Furthermore, Rac1 and Mapk14 connected leukocyte transendothelial migration pathway with the pathways of colorectal cancer, renal cell carcinoma, osteoclast differentiation. The pathway of complement and coagulation cascades was connected to phagosome by MBL2. Rac1, as a key knot molecule, links the above pathways into an immune defense network.

### Validation of DEPs in serum by ELISA

**Fig. 7:**
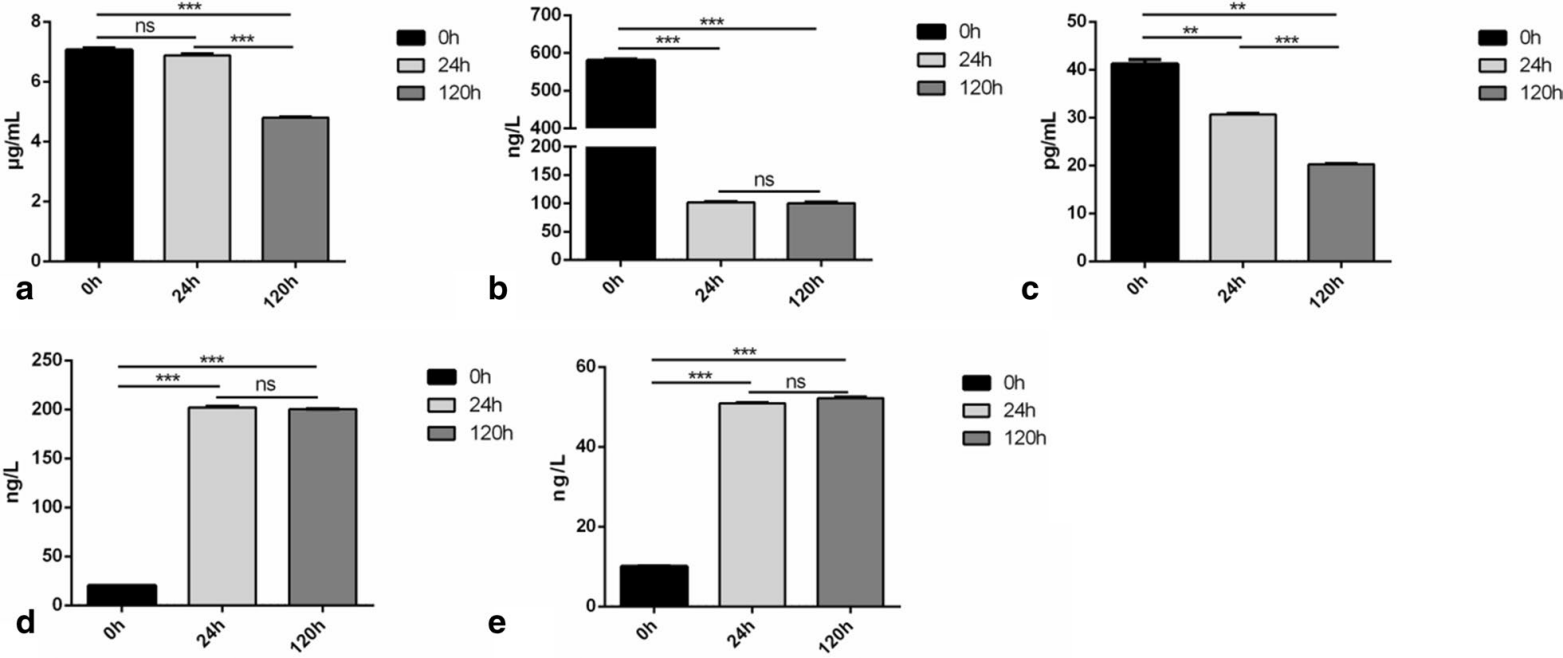
Validation of DEPs by ELISA

To validate the iTRAQ results, DEGs related to endocytosis and inflammatory responses in serum which were found dramatically changed were chosen to test by ELISA kits. We measured the expression levels of five proteins, including up- and down-regulated, the data had the same trend as for the iTRAQ results. Down-regulated levels were found for CFB(Complement factor B), ITGB(Integrin beta) and E-cadherin (Fig. 7a–c). For CFB, progressive decreasing concentrations in serum were observed at 120 h while E-cadherin declined from 24 h. ITGB showed a gradual descent after APP infection. Up-regulated levels were found for MRC1(Macrophage mannose receptor C type-1) and PRDX2 (Peroxiredoxin 2) following experimental infection with APP (Fig. 7d, e). These two proteins showed an increased tendency after infection at 24 h and 120 h.

## Discussion

In this study, a pneumonia model of APP-infected piglets was successfully established, and the serum of piglets from different infection stages was investigated using iTRAQ-LC–MS/MS technology. We found that APP could delay host inflammatory response to the infection at early stage, and then induce innate immunity to convert from adhesion, interaction into complement activation, proteasome digestion, bacterial invasion, followed by systemic tissue immune injury. This would increase our understanding of the porcine distinct response to APP infection. These results not only update the characteristic analysis of responses in infected hosts to APP, but also provide a new direction for understanding host natural and adaptive immunity.

Although proteomic methods have made great progress, there are few big data to reveal the changes of piglets infected with APP by proteomic techniques. Up to now, there were only two proteomic studies to take hosts as the research object about APP. In 2006, Hennig-Pauka et al. used 2DE and mass spectrometry to analyze proteins in bronchoalveolar lavage fluid (BALF) of piglets infected with APP. Antimicrobial peptides, prophenin-2 and PR-39, and the calcium-binding granulin C were found up-regulated in piglets with chronic infection (Hennig-Pauka et al. 2006). PR-39 was significantly up-regulated in BALF, but not in serum within 21 days after infection, suggesting that PR-39 may be a potential diagnostic marker. In 2009, Miller et al. used 2DE technology to identify species-specific proteins in piglet serum, such as IgM and major acute phase proteins, which are different from those in other mammals (Miller et al. 2009). With the location characteristics of these proteins in 2D electrophoresis as reference spectra, PCV2 and APP as examples, they confirmed that the host would change the characteristic spectra after infected with pathogens. It suggests that this method may be of reference value in the diagnosis of infection. There was only one paper took APP as the research object. In 2006, Liao et al. screened 30 immunogenic structure proteins by Western blot analysis after electrophoresis of APP outer membrane proteins and extracellular proteins (Liao et al. 2009).

In our study, the iTRAQ technique with much higher sensitivity than 2DE was used. High throughput screening obtained abundant differential proteins in the serum of piglets infected with APP. For the first time, the interference of APP infection on the innate immunity, physiological and metabolic activities of the host and the response of the host were comprehensively analyzed, and some important natural immune molecules participants in the body’s resistance to APP infection were found, such as Complement Component, Peroxiredoxin 2, Macrophage mannose receptor C-type-1. These proteins tend to rise throughout the course of the infection. By analyzing the plasma proteomics data, Ye Lu et al. found that peroxidase 2 could be used as a diagnostic marker for early hepatitis b virus infection (Lu et al. 2010). After *Vibrio anguillarum* (Ren et al. 2013) and hemophilic influenza (Murphy et al. 2005) infection, peroxidase 2 rose significantly, which is consistent with our data. Min-kyoung et al. found that MRC1 significantly gone up after *Mycobacterium avium* subsp. paratuberculosis infection (Shin et al. 2015).Notably, enrichment of tuberculosis-associated signaling pathways was also found in our data after APP infection. This suggests a new mechanism of host immune response after APP infection, and also provides a basis for APP diagnosis of potential target proteins. The changes in serum during the different stages of APP infection displayed characteristics of defense immunity. We found, that piglet serum showed changes in immune inflammatory responses.

In particular, pathway and network analysis data also showed that the complement and coagulation cascades played an important role in the host response. Yin et al. found that there was an increase in complement and coagulation cascades after cryptocaryoniasis infection and the alternative way played an important role at the early stage which result to immune suppression (Yin et al. 2016). Lin et al. discovered that in the co-infection of Swine influenza and *Streptococcus suis* there could also indicate that complement and coagulation cascades may be a marker of the serious inflammation and might predict severe respiratory syndrome(Lin et al. 2015).We detected CFB,a protein related to complement and coagulation cascades. The decrease of CFB at the early stage may lead to the incomplete function of macrophages, which is consistent with the negative regulation of immunity of APP to the host. In the late stage of infection the related molecules of complement and coagulation cascades changed to phagosome, focal adhesion and leucocyte migraton, which indicated that host’s immunity had transmitted from primary to adaptive. In the early stage of infection, “S24-VS-S0” showed an overall upward trend, and after 24 h, a large proportion of them showed a downward trend. Complement is an important way for the body to fight infection and plays an important role in the absence of adaptive immunity. The complement system consists of serum and cell surface proteins, which play crucial roles in the immune system (Bandara et al. 2003). Activation of the complement and coagulation cascades is a key event for the efficient response of the immune system (Ward and Inzana 1994). The functions of complement, proteins in serum include phagocytosis, pathogen killing, immune complex clearance, inflammatory responses, and antibody production (Ward and Inzana 1994). Our study suggested that APP affect the induction of host immunoregulatory proteins and inhibit neutrophil chemotaxis in the early stage, while the inflammatory response was extremely active for clearing bacteria at the late stage (120 h), such as T cell activation and STAT3 phosphorylation activation. This provides rich content for us to study the pathogenic mechanism of APP, and may be helpful for the development of diagnostic agents and therapeutic drugs.

The amoebiasis and endocytosis pathways were significantly enriched at 120 h. The amoebiasis pathway could lead production to reactive oxygen species and mediators of neutrophil apoptosis and lung damage (Moonah et al. 2013). Endocytosis pathways regulate TLR4 signaling and link innate and adaptive immunity. The LPS receptor complex (TLR4 and CD14) and an associated protein (MD-2) not only signal the presence of the microbes but direct the endocytosis of antigens on class II molecules for presentation to Th cells (Kuzmich et al. 2017). The data suggest bridging of innate and adaptive immunity with time.

CD14, which is expressed mainly on the surface of macrophages, neutrophils, and interstitial dendritic cells, belongs to the group of pattern recognition receptors (Brogaard et al. 2015). It recognizes structural components of bacteria (termed pathogen-associated molecular patterns, or PAMPs) during the first steps of infection. Membrane-bound CD14 acts as a receptor for LPS combined with LPS-binding protein (Schmitz and Orso 2002). The recognition of APP components by CD14 triggers a series of events. Our results suggest that the host attempted to clear APP infection, which was associated with an upregulation of CD14 expression. The increased expression of CD14 may be related to excessive inflammation of the host immune response. Thus, CD14 could be a potential target for the regulation of excessive inflammation elicited by APP. We found CD14 as a central molecule through the pathways and networks analysis. CD14 is a kind of programmed recognition receptor and distributes on the surface of some immune cells like macrophage, neutrophil and so on. CD14 can recognize the LPS of bacteria and induce the production of some cytokines such as TNF-α (Wright et al. 1990). Yersinia app. depends on a CD14 manner leading to immunosuppression by IL-10 (Sing et al. 2002).

Meanwhile, we also found some down-regulated immune-related molecules and pathways after APP infection, such as cell adhesion. We examined E-cadherin, a cadherin protein that plays an important role in regulating and maintaining the integrity of cellular connections. Man found that adenovirus replication in tracheal epithelial cells increased significantly after the use of calcitonin antibodies (Man et al. 2015) and Chan et al. found that when *H. pylori* was infected, e-cadherin would undergo significant methylation and lead to the expansion of the infection range (Chan et al. 2006).These suggest that the pathogen infection will affect the host’s e-cadherin and cause damage to the body. The descending signaling pathway also involves endocytosis and T cell homeostasis. We speculate that in the late stage of infection, host adaptive immune response gradually begins to play a role, leading to a decrease in endocytosis, while T cell homeostasis is temporarily out of balance due to adaptive immune response and is activated to play a role.We also detected another protein, integrin, which was down-regulated during infection. Integrins are the main family of receptors on the cell surface, mediating the adhesion of cells to the extracellular matrix (ecm), which plays a key role in the WBC migration, platelet aggregation and wound healing. Failure to achieve integrin-mediated adhesion may result in cell death. And integrin falls suggests that it might be a mechanism that causes injury.

Investigations are necessary to reveal the interaction mechanism between the host and APP and find potential biomarkers for diagnosis. In the future, we will explore the roles of these signaling pathways and the DEPs related to the pathogenesis of APP infection and immune responses to clarify the interaction between the host and APP.

In a conclusion, hundreds of DEPs were identified at different stages during APP infection and these proteins have functions linked with the extracellular exosome, focal adhesion, and other biological processes at an early stage; whereas complement and coagulation cascades, immunological synapse, inflammatory response, endocytosis, and bacterial invasion of epithelial cells were significantly enhanced at the late stage. The complement and coagulation cascades play an important role in the process of the host response; furthermore, CD14 was the crucial crosstalk molecule in multi pathways after APP infection condition. These findings increase our understanding of the dynamics of host immune response during APP infection.

## Supplementary information

**Additional file 1: Table. S1.** Representative up-regulated proteins in the serum of the “S24-Vs-S0” stage with a 2.0-fold change.

**Additional file 2: Table. S2.** Representative up-regulated proteins in the serum of the “S120-Vs-S0” stage with a 2.0-fold change.

**Additional file 3: Table. S3.** Representative up-regulated proteins in the serum of the “S120-Vs-S24” stage with a 2.0-fold change.

**Additional file 4: Table. S4.** Representative down-regulated proteins in the serum of the “S24-Vs-S0” stage with a 2.0-fold change.

**Additional file 5: Table. S5.** Representative down-regulated proteins in the serum of the “S120-Vs-S0” stage with a 2.0-fold change.

**Additional file 6: Table. S6.** Representative down-regulated proteins in the serum of the “S120-Vs-S24” stage with a 2.0-fold change.

## Data Availability

The data on which the conclusions are made are all presented in this paper. All of raw data can be found on PRIDE (https://www.ebi.ac.uk/pride/archive) with an identifier number PXD017500

## Abbreviations

APP: *Actinobacillus pleuropneumoniae*
iTRAQ: Isobaric tags for relative and absolute quantitation
LC–MS/MS: Liquid chromatography-tandem mass spectrometry
DEPs: Differentially expressed proteins
PAMPs: Pathogen-associated molecular patterns
