# Supplementary material for: iTRAQ-based quantitative proteomic analysis of peripheral blood serum in piglets infected with Actinobacillus pleuropneumoniae
Source: AMB Express. 2020 Jul 6;10:121. doi: 10.1186/s13568-020-01057-9 (PMC7338327; doi:10.1186/s13568-020-01057-9)
Supplement: Supplementary file 1 — Additional file 1: Table. S1. Representative up-regulated proteins in the serum of the “S24-Vs-S0” stage with a 2.0-fold change. [file 13568_2020_1057_MOESM1_ESM.doc]

| **Protein name** | **Protein ID** | **Gene Name** |
| --- | --- | --- |
| **Immunologic proteins** |  |  |
| IgG heavy chain | L8B0W5_PIG | IGHG |
| Complement component C7 | F1SMJ1_PIG | C7 |
| Mannose-binding protein C | MBL2_PIG | MBL2 |
| Macrophage mannose receptor C type-1 | G9K122_PIG | MRC1 |
| Macrophage colony-stimulating factor 1 receptor | K9IVS4_PIG | CSF1R |
|  |  |  |
| **Physiologic proteins** |  |  |
| Heat shock protein HSP 90-alpha | HS90A_PIG | HSP90AA1 |
| Vitronectin | I3LP50_PIG | VTN |
| Fibroblast growth factor | I3L7I1_PIG | FGF11 |
| Peroxiredoxin 2 | F1SDX9_PIG | PRDX2 |
| Glutathione S-transferase P | GSTP1_PIG | GSTP1 |
| Ubiquitin-conjugating enzyme E2L 3 | B8Y648_PIG | UBE2L3 |
|  |  |  |
| **Acute-phase proteins** |  |  |
| Pentaxin | F1RJ76_PIG | CRP |
| Transforming growth factor beta-2 | TGFB2_PIG | TGFB2 |
| Fibroblast growth factor | I3L7I1_PIG | FGF11 |
| Elongation factor 1-gamma | EF1G_PIG | EEF1G |
| Serum amyloid A protein | F1S9C0_PIG | SAP |
