# Supplementary material for: iTRAQ-based quantitative proteomic analysis of peripheral blood serum in piglets infected with Actinobacillus pleuropneumoniae
Source: AMB Express. 2020 Jul 6;10:121. doi: 10.1186/s13568-020-01057-9 (PMC7338327; doi:10.1186/s13568-020-01057-9)
Supplement: Supplementary file 3 — Additional file 3: Table. S3. Representative up-regulated proteins in the serum of the “S120-Vs-S24” stage with a 2.0-fold change. [file 13568_2020_1057_MOESM3_ESM.doc]

| **Protein name** | **Protein ID** | **Gene Name** |
| --- | --- | --- |
| **Immunologic proteins** |  |  |
| Lactoferrin (Fragment) | Q8WMN8_PIG | LTF |
| Peptidoglycan-recognition protein | Q6PKY6_PIG | PGLYRP |
| Transforming growth factor-beta-induced protein ig-h3 | F1RHA7_PIG | TGFBI |
| IgG heavy chain | L8B0Y6_PIG | IGHG |
| Vascular cell adhesion molecule | Q29123_PIG | VCAM |
| Macrophage colony-stimulating factor 1 receptor | K9IVS4_PIG | CSF1R |
| Platelet endothelial cell adhesion molecule | PECA1_PIG | PECAM1 |
|  |  |  |
| **Physiologic proteins** |  |  |
| Calcium-activated chloride channel regulator 1 | F1S4C9_PIG | CLCA1 |
| Serum amyloid A protein | F1S9B8_PIG | LOC100526034 |
| Matrix metallopeptidase 9 | Q2VI03_PIG | MMP9 |
| Cystatin | Q0Z8R0_PIG | CST3 |
| Insulin-like growth factor-binding protein 2 | I3LQ27_PIG | IGFBP2 |
| Lipoprotein lipase | Q6J9X9_PIG | LPL |
| Calcium-binding protein A9 | C3S7K6_PIG | S100A9 |
| Aldose reductase | A0A140TAK7_PIG | AKR1B1 |
