# Supplementary material for: iTRAQ-based quantitative proteomic analysis of peripheral blood serum in piglets infected with Actinobacillus pleuropneumoniae
Source: AMB Express. 2020 Jul 6;10:121. doi: 10.1186/s13568-020-01057-9 (PMC7338327; doi:10.1186/s13568-020-01057-9)
Supplement: Supplementary file 4 — Additional file 4: Table. S4. Representative down-regulated proteins in the serum of the “S24-Vs-S0” stage with a 2.0-fold change. [file 13568_2020_1057_MOESM4_ESM.doc]

| **Protein name** | **Protein ID** | **Gene Name** |
| --- | --- | --- |
| **Immunologic proteins** |  |  |
| Ficolin-1 | I3LGI8_PIG | FCN1 |
| Lysozyme C-1 | LYSC1_PIG | Lyz1 |
| Transforming growth factor beta-2 | TGFB2_PIG | TGFB2 |
| Elongation factor 1-gamma (Fragment) | EF1G_PIG | EEF1G |
|  |  |  |
|  |  |  |
| **Physiologic proteins** |  |  |
| Apolipoprotein M | A5D9L6_PIG | APOM |
| Creatine kinase M-type | I3LBJ8_PIG | CKM |
| Peptidyl-prolyl cis-trans isomerase | I3LUC8_PIG | PPIC |
| Pyruvate kinase | F1SHL9_PIG | PKM |
| Glucose-6-phosphate isomerase | F1RNU9_PIG | GPI |
| Aspartate aminotransferase, cytoplasmic | AATC_PIG | GOT1 |
| Mitogen-activated protein kinase | F1RYA1_PIG | MAPK14 |
