# Supplementary material for: iTRAQ-based quantitative proteomic analysis of peripheral blood serum in piglets infected with Actinobacillus pleuropneumoniae
Source: AMB Express. 2020 Jul 6;10:121. doi: 10.1186/s13568-020-01057-9 (PMC7338327; doi:10.1186/s13568-020-01057-9)
Supplement: Supplementary file 5 — Additional file 5: Table. S5. Representative down-regulated proteins in the serum of the “S120-Vs-S0” stage with a 2.0-fold change. [file 13568_2020_1057_MOESM5_ESM.doc]

| **Protein name** | **Protein ID** | **Gene Name** |
| --- | --- | --- |
| **Immunologic proteins** |  |  |
| Integrin beta (Fragment) | D6BR76_PIG | ITGB |
| Endoplasmin | F1SRK6_PIG | HSP90B1 |
| C1q and tumor necrosis factor-related protein 3 isoform a | B2LWN5_PIG | C1QTNF3 |
| Complement factor B (Fragment) | CFAB_PIG | CFB |
|  |  |  |
| **Physiologic proteins** |  |  |
| Tyrosine-protein kinase | F1S1L0_PIG | BTK |
| Coagulation factor XIII, A1 polypeptide | K7GQL2_PIG | F13A1 |
| Triosephosphate isomerase | D0G7F6_PIG | TPI1 |
| GTP-binding nuclear protein Ran | F1RFQ7_PIG | RAN |
| Insulin-like growth factor binding protein 6 | A6ZIC9_PIG | IGFBP6 |
| Epidermal growth factor receptor (Fragment) | Q9N0K4_PIG | egf |
| Alpha-fetoprotein | FETA_PIG | AFP |
