# Supplementary material for: iTRAQ-based quantitative proteomic analysis of peripheral blood serum in piglets infected with Actinobacillus pleuropneumoniae
Source: AMB Express. 2020 Jul 6;10:121. doi: 10.1186/s13568-020-01057-9 (PMC7338327; doi:10.1186/s13568-020-01057-9)
Supplement: Supplementary file 6 — Additional file 6: Table. S6. Representative down-regulated proteins in the serum of the “S120-Vs-S24” stage with a 2.0-fold change. [file 13568_2020_1057_MOESM6_ESM.doc]

| **Protein name** | **Protein ID** | **Gene Name** |
| --- | --- | --- |
| **Immunologic proteins** |  |  |
| Transmembrane secretory component poly-Ig receptor (Fragment) | A0A0E3M2Q4_PIG | TSCPR |
| Complement component C7 | F1SMJ1_PIG | C7 |
| Heat shock protein HSP 90-alpha | HS90A_PIG | HSP90AA1 |
| Platelet glycoprotein Ib beta | B7TY21_PIG | GPIbB |
| Complement component C8B | A0SEH2_PIG | C8 |
| Caspase-3 | CASP3_PIG | CASP3 |
|  |  |  |
| **Physiologic proteins** |  |  |
| Tyrosine-protein kinase | F1S1L0_PIG | BTK |
| Coagulation factor XIII, A1 polypeptide | K7GQL2_PIG | F13A1 |
| E-cadherin | C6EVT4_PIG | ECA |
| Rab GDP dissociation inhibitor beta | F1RUK8_PIG | GDI2 |
| Secreted phosphoprotein 1 | D0G7G0_PIG | SPP1 |
| Chloride intracellular channel protein 1 (Fragment) | CLIC1_PIG | CLIC1 |
| Tubulin beta chain | TBB5_PIG | TUBB |
| Pyruvate kinase | F1SHL9_PIG | PKM |
| Superoxide dismutase 1 (Fragment) | Q95ME5_PIG | SOD1 |
| Cell division control protein 42 homolog | CDC42_PIG | CDC42 |
